# Effects of Remedial Sport Hunting on Cougar Complaints and Livestock Depredations

**DOI:** 10.1371/journal.pone.0079713

**Published:** 2013-11-19

**Authors:** Kaylie A. Peebles, Robert B. Wielgus, Benjamin T. Maletzke, Mark E. Swanson

**Affiliations:** 1 Large Carnivore Conservation Laboratory, School of Environment, Washington State University, Pullman, Washington, United States of America; 2 Washington Department of Fish and Wildlife, Olympia, Washington, United States of America; 3 School of Environment, Washington State University, Pullman, Washington, United States of America; Panthera, United States of America

## Abstract

Remedial sport hunting of predators is often used to reduce predator populations and associated complaints and livestock depredations. We assessed the effects of remedial sport hunting on reducing cougar complaints and livestock depredations in Washington from 2005 to 2010 (6 years). The number of complaints, livestock depredations, cougars harvested, estimated cougar populations, human population and livestock populations were calculated for all 39 counties and 136 GMUs (game management units) in Washington. The data was then analyzed using a negative binomial generalized linear model to test for the expected negative relationship between the number of complaints and depredations in the current year with the number of cougars harvested the previous year. As expected, we found that complaints and depredations were positively associated with human population, livestock population, and cougar population. However, contrary to expectations we found that complaints and depredations were most strongly associated with cougars harvested the previous year. The odds of increased complaints and livestock depredations increased dramatically (36 to 240%) with increased cougar harvest. We suggest that increased young male immigration, social disruption of cougar populations, and associated changes in space use by cougars - caused by increased hunting resulted in the increased complaints and livestock depredations. Widespread indiscriminate hunting does not appear to be an effective preventative and remedial method for reducing predator complaints and livestock depredations.

## Introduction

Sport hunting is often used as a preventative or remedial measure to reduce carnivores and related human complaints and/or livestock depredations for many predators including, brown bears (*Ursus arctos arctos*) [1], cougars (*Puma concolor*) [2], grizzly bears (*Ursus arctos horribilus*) [3], jaguars (*Panthera onca*) [4], leopards (*Panthera pardus*) [5], lions (*Panthera leo*) [6], and others [7]. However, to our knowledge, the assumption that increased sport hunting reduces complaints and depredations has not been scientifically tested as yet [7].

For example, cougars (our model animal) have one of the broadest distributions of any mammal in the Western Hemisphere with a range that includes much of the North and South American continents [8]. This large, solitary carnivore is highly adaptable and occupies a wide variety of habitats [9]. Following European colonization of the Americas, their populations and range were diminished due to extensive harvest and population control through bounties - because cougars were often viewed as unacceptable threats to life and property [8].

After the bounty era ended cougars were still often viewed as potential threats to life and property. This view led to state management plans in the United States that were focused on reducing cougar populations to decrease cougar-human interactions primarily through increased sport hunting [10]. Many of these management plans based their cougar population estimates and harvest objectives solely (e.g. Washington Department of Fish and Wildlife until 2012) or in part on the number of complaints and depredations [10], [11], [12], [13], [14]. In Washington, as the number of complaints increased, the hunter effort and opportunity increased through lengthened seasons and higher bag limits - in response to what was thought to be a rapidly growing cougar population [10].

However, contrary to the public perception of increasing cougar populations, several areas with increasing numbers of complaints and depredations corresponded with declining female cougar populations and increasing male populations [2], [15]. Heavy hunting (>25% per year) caused the female population growth rate to decline [2], [15]. However, compensatory immigration [15] and emigration [16] by mostly males resulted in a stable observed growth rate with no net change in total cougar population size. Heavy remedial hunting of cougars simply changed the population age-sex structure towards younger immigrant male cougars in a source-sink dynamic [16]. The same phenomenon of increased male immigration and female decline with no net change in total numbers following increased hunting was also observed in grizzly bears populations [17], [18], [19]. These results suggest that remedial sport hunting might not reduce cougar (and other carnivore) populations and associated complaints and livestock depredations. In this paper we test the widely accepted hypothesis that increased sport hunting will decrease cougar complaints and depredations in a large scale (statewide) long-term (6 years) observational experiment. The “remedial hunting” hypothesis predicts that complaints and livestock depredations will decrease following increased sport hunting. The “source-sink” hypothesis predicts that complaints and livestock depredations will remain stable, or even increase [2], following increased sport hunting.

## Methods

### Study Area

The state of Washington encompasses approximately 172,111 km^2^ with natural regions ranging from a sea level coastal temperate rainforest to the Cascade mountain range to the Palouse prairie [20]. Cougars inhabited approximately 61% of the land mass of the state [21].

The Cascade Range reaches elevations of 4,395 m and divides the state into two distinct climate regions. The areas west of the Cascades have a temperate maritime climate characterized by mild wet winters and cool summers [22]. Average temperatures in the western regions of Washington range from 0°C in January to above 16°C in July. The areas east of the Cascade mountain range have a much drier climate with hot summers and much colder winters. Average temperatures in eastern Washington range from −18°C in January to 32°C in July. Forest vegetation covers approximately 51% of the total land area of Washington with the majority of forested regions located in the mountainous sections of Western and Northeastern Washington [22].

### Data Collection

We collected data on numbers of people and numbers of cougars because these should be positively related to numbers of complaints. We also collected data on numbers of livestock and numbers of cougars because these should be positively related to numbers of depredations.

Finally, we collected data on numbers of cougars killed because these should be negatively related to the number of both complaints and depredations, according to the remedial hunting hypothesis.

### Complaints and Depredations

We obtained the total number of cougar complaints from the Washington Department of Fish and Wildlife’s Cougar Incident Database and categorized them based on the confidence level determined by agency staff (verified, possible, and unlikely). Verified cougar complaints and depredations were investigated and confirmed by Washington Department of Fish and Wildlife (WDFW) officers and only verified complaints were used in this analysis. Possible and unlikely complaints were not investigated or confirmed by WDFW officers and thus were not used in the analysis because those types of complaints (phone calls, verbal reports) could not be verified and appeared to be driven by socio-political, not biological factors [21], [23]. Depredation events consisted of attacks or killings of domestic livestock and pets (*Canis lupus familiaris, Felis catus*) confirmed by WDFW officers. We refer to all depredations on domestic animals as “livestock depredations.” We compiled the tallies for all 39 counties and 136 GMUs, in Washington for the six year time series (2005–2010), and removed all blank and duplicate cougar complaints.

### Cougar Populations

We estimated the expected cougar population size for each county and GMU (game management unit) using an adult density of 1.7/100 km^2^ and a total density of 3.5/100 km^2^ for all cougar habitat in Washington [21]. These estimates are for animals >2 years of age and were based on long term (1998–2013) replicated (6 study areas) research studies throughout the state, which showed little or no variation in density regardless of location, time or level of harvest [16], [21].

### Human Population

The number of people in each county and GMU during each year was obtained from the United States Census Bureau Quick Facts (2010). We converted the census data from census block polygons into centroids with the number of people per census block [23]. We then used a spatial join in ArcMap 9.3 to determine the number of people per GMU and calculated density by dividing by the area of each GMU (GMU mean area = 1232.62 km^2^, standard deviation = 1103.55 km^2^).

### Livestock Numbers

The numbers of livestock were obtained from the United States Department of Agriculture National Agricultural Statistics Service for each county in Washington during 2005–2010 [24]. We tallied the livestock numbers and placed them into two categories for each county: large or deer-sized livestock and small livestock. The category for large or deer sized livestock consisted of alpacas (*Vigugna pacos*), llamas (*Lama glama*), cattle (*Bos primigenius*), equine (*Equus caballus*), goats (*Capra aegagrus*), hogs (*Sus scrofa*) and sheep (*Ovis aries*). Small livestock consisted of chickens (*Gallus gallus domesticus*), ducks (family Anatidae), geese (genus *Anser*), pheasants (*Phasianus colchicus*), and turkeys (*Meleagris gallopavo*). The numbers of livestock across the state were only available in summary form for each county and the boundaries were not consistent with GMUs- so we could only use livestock in the county-level analysis.

### Cougars Harvested

We obtained the number of cougars harvested through sport harvest in each GMU each year from the Washington Department of Fish and Wildlife’s Game Harvest Report Database (http://wdfw.wa.gov/hunting/harvest/). The numbers of cougars harvested across the state were only available by GMU and the boundaries were not consistent with the county boundaries so we could not use harvest in the county level analysis.

Because cougar harvest management is based on adult (>2 year old) density (1.7/100 km^2^) in Washington (WA) [21], we calculated the proportion of cougars harvested in each GMU by taking the number of cougars harvested by sport hunters divided by the number of adult cougars estimated to be on the landscape for that GMU. We did not analyze the effects of depredation removals by WDFW personnel separately, because such livestock depredations were handled by issuing additional hunting permits to the landowner (allowing the use of tracking hounds) in response to the depredation [10].

### Data Analysis

#### Statistical analysis

We used a negative binomial general linearized model to assess the relationship between verified reports and county- and GMU-level factors. The negative binomial error distribution was used rather than a Poisson error distribution to analyze our frequency data (complaints, depredations) because our dependent variables consisted of 0 to positive integer count data with a variance exceeding the mean [25]. A negative binomial general linearized model is appropriate for this type of over-dispersed count data with numerous zeros. We also tested a zero-inflated negative binomial model, which estimates regression coefficients for two components: one modeling the response variable with a negative binomial distribution, and one component accounting for a disproportionate occurrence of zero values in the model [26]. However, goodness-of-fit tests indicated that the additional fitting precision associated with this method was unnecessary. The most appropriate statistical model was then selected using the AIC (Akaike Information Criterion) and log-likelihood values [27]. The rate ratio, analogous to odds-ratio, was computed from the coefficients to aid in interpreting the results [28]. For example, a rate ratio of 1.0 for any independent variable means the effect on the dependent variable is unchanged. A rate ratio of 1.5 means the odds are increased by 50%, a ratio of 2.0 means the odds are increased by 100% etc. Descriptive statistics for all variables and negative binomial regression models were generated for verified complaints, verified livestock depredations, and verified total depredations using the R environment for statistical programming [29].

#### County-based tests

The independent variables obtained from county data were human population, livestock numbers, and number of cougars. Complaints and depredations were the dependent variables. To determine which variables have a statistically significant relationship with cougar complaints and depredations we used a negative binomial generalized linear model (coefficients tested at α = 0.05).

#### GMU-based tests

The independent variables obtained from GMU were number of cougars, number of cougars harvested, proportion of cougars harvested and human population. The number of livestock was not available by GMU, but comparing the odds ratio between the county and the GMU level tests allows for direct comparison of the relative effects of livestock compared to the other independent variables. For example, if the odds of a livestock depredation are increased from 1 to 1.5 with each additional livestock, and the odds of a depredation are increased from 1 to 2.5 with each additional cougar, we can conclude that the number of cougars has a larger effect than additional livestock on the probability of livestock depredations. To determine which factors have a statistically significant relationship with cougar reports and depredations we used a negative binomial generalized linear model (coefficients tested at α = 0.05). In order to establish directionality of putative causation, we used the previous year’s harvest and the following year’s cougar complaints or depredations to determine statistically significant relationships. Cougar complaints and depredations were the dependent variables. We also tested for the effects of the previous 2–4 year time-lagged harvest, but those results are not reported here because they were almost identical to the 1 year time-lagged data presented here.

## Results

### County-based Tests

The total number of non-duplicated complaint reports between January 2005 and May 2010 was 2648; 432 reports were verified and 166 of those verified complaints were livestock depredations. Over the course of the 6-year time series, the number of total and verified cougar complaints generally declined while depredations remained relatively constant (Table 1,2). For a distribution map of reports by county across the state see supporting Figure S1.

**Table 1:** Total reports collected for all 39 counties in Washington between Jan. 2005–May 2010.

Year	Verified Reports	Total Reports	Livestock Depredation	Total Depredation
**2005**	114	743	28	38
**2006**	88	581	32	42
**2007**	73	418	27	37
**2008**	63	408	30	34
**2009**	63	426	36	39
**2010**	31	110	13	19

**Table 2:** Basic descriptive statistics for county-level data from Washington, 2005–2010. Statistics shown are for the number of reports in each county for each year.

Factor	Minimum	Maximum	Range	ArithmeticMean	StandardError	95% ConfidenceInterval	StandardDeviation
**Verified Reports**	0	28	28	1.846	0.211	1.429–2.263	3.235
**Livestock Depredations**	0	11	11	0.709	0.105	0.503–0.916	1.602
**Total Depredations**	0	12	12	0.889	0.122	0.648–1.130	1.870
**Population**	2091	1931249	1929158	166894.551	21461.009	124612.122–209176.981	328290.305
**Habitat (km^2^)**	190.447	11357.910	11167.463	2679.532	150	2384.002–2975.062	2294.562
**Deer Sized Livestock**	1549	139244	137695	18925.333	1555.954	15859.796–21990.871	23801.526
**Small Sized Livestock**	20	1510438	1510418	61626.205	16455.393	29205.828–94046.582	251719.109

The county-based model revealed that the primary factors influencing verified complaints were the year and total expected cougar population (Table 3, Results S1). However, each additional cougar on the landscape only increased the odds of a verified complaint by 1.00847 times or approximately 1%.

**Table 3:** Summary of best county-level model outputs.

Dependent variable	IndependentVariable	Estimated Coefficient	Null Deviance	ResidualDeviance	AIC	Standard Error
Verified Reports	Year	−0.248	337.30	228.09	761.68	0.178
	Cougar population	0.0084				
Livestock Depredations	Human population	1.789×10^−2^	226.31	162.28	476.86	0.139
	Cougar population	4.36×10^−2^				
	Large livestock	2.336×10^−4^				
Total Depredations	Human population	1.583×10^−2^	258.05	176.97	533.53	0.159
	Cougar population	4.137×10^−2^				
	Large livestock	2.176×10^−4^				

Several variables also influenced the number of livestock depredations at the county level including human population, the number of large livestock, and the total cougar population on the landscape. As the human population increased in an area the number of livestock depredations also increased in that area. With each increase in 10,000 people in an area the probability of a livestock depredation occurring in that area increased by 1.018 times or approximately 2%. For each additional 2000 large livestock in the area the chance of a livestock depredation occurring increased by 1.0002 times or less than 1%. For each additional cougar on the landscape the chance of a livestock depredation occurring increased by 1.0446 times or approximately 5%. For each additional 2000 large livestock in the area the chance of a livestock depredation occurring increased by 1.0002 times or less than 1%.

The final county-level model analyzed possible factors that influence the number of total verified depredations (livestock and pets). This model revealed that human population, the number of large livestock, and total cougar population present all are correlated with the number of depredations (Table 3). With each increase in 10,000 people in an area the probability of a depredation occurring in that region increased by 1.016 or approximately 2%. For each additional livestock animal the probability of a depredation being reported increased by 1.00022 times or less than 1%. For each additional cougar present the chance of a depredation occurring in that area increased by 1.042 or 4%.

Overall, the effects of numbers of people, livestock and cougars on the odds of total reports, verified reports, livestock depredation and total depredations were marginal, averaging from 1% to 5%.

### GMU-based Tests

The total number of non-duplicated complaints between January 2005 and May 2010 was 2647; 429 complaints were verified and 166 of those verified complaints were livestock depredations. Over the course of 6 years the number of total and verified complaints generally declined while depredations remained relatively constant (Table 4). Descriptive statistics for all variables tested were also generated in statistical program R (Table 5). For the distribution of reports across the state by GMU see supporting Figure S2.

**Table 4:** Total reports collected for all 136 GMUs in Washington from January 2005 to May 2010.

Year	Verified Reports	Total Reports	Livestock Depredation	Total Depredation	Cougars Harvested
**2005**	111	674	28	37	182
**2006**	86	569	32	41	199
**2007**	72	416	28	38	198
**2008**	61	398	28	31	188
**2009**	63	416	37	40	140
**2010**	30	106	13	19	161

* 107 total reports and 9 verified reports removed because no GMU was listed in the complaint.

**Table 5:** Basic descriptive statistics for GMU-level tests. Statistics shown are for each GMU for each year.

Factor	Minimum	Maximum	Range	ArithmeticMean	StandardError	95% ConfidenceInterval	StandardDeviation
**Verified Reports**	0	11	11	0.526	0.042	0.443–0.608	1.197
**Livestock Depredations**	0	9	9	0.203	0.025	0.155–0.252	0.708
**Total Depredations**	0	10	10	0.255	0.027	0.201–0.309	0.782
**Cougars Harvested**	0	15	15	1.331	0.077	1.180–1.482	2.194
**Habitat (km^2^)**	2.759	2713.761	2711.003	667.545	19.033	630.185–704.904	543.689
**Proportion of Adult Cougars Harvested**	0.000	1.9101	1.9100	0.117	0.007	0.103–0.132	0.210

Two models were selected for determining which factors are related to the number of verified complaints in each GMU (Table 6, Results S1). The first model was g(y) = −1.970170+0.308764 (number of cougars harvested) +0.031093 (total cougar population) –0.003842 (cougars harvested*total cougar population).

**Table 6:** Summary of best GMU-level model outputs.

Dependent Variable	Independent Variable	EstimatedCoefficients	NullDeviance	ResidualDeviance	AIC	StandardError
Verified Reports	Cougars harvested	0.308	496.17	422.43	1123.1	0.0697
	Cougar population	0.031				
Verified Reports	% cougars harvested	9.57×10^−1^	444.32	416.63	1157.1	0.0510
	Human population	1.066×10^−6^				
Livestock Depredations	Cougars harvested	0.428	310.00	253.63	644.87	0.0561
	Cougar population	0.038				
Livestock Depredations	% cougars harvested	1.216	268.75	247.24	668.72	0.0377
	Human population	1.278×10^−6^				
Total Depredations	Cougars harvested	0.386	360.63	295.05	743.66	0.0647
	Cougar population	0.038				
Total Depredations	% cougars harvested	9.633×10^−1^	310.50	288.64	775.32	0.0421
	Human population	1.164×10^−6^				

The number of cougars harvested was positively related to the number of verified complaints per GMU (rate ratio = 1.36174, *z* = 5.081, *P*<0.001). For each additional adult cougar harvested during the previous year the odds of a complaint increased by 1.36174 or 36%. The total expected population of cougars was also found to be positively associated with increased numbers of verified complaints (rate ratio = 1.03158, z = 5.819, *P*<0.001). For each additional cougar on the landscape the odds of a verified complaint being filed increased by 1.03158 or 3%. The effect of cougars harvested the previous year on the odds of verified complaints is 10 times higher (1.36 vs 1.03) than the effect of number of cougars on the landscape.

The second model selected for determining which factors may influence the number of verified complaints per GMU was g(y) = −1.081+0.9571 (proportion of adult cougars harvested) +1.066×10^−6^ (human population) +1.453×10^−5^ (proportion of adult cougars harvested*human population).

The proportion of adult cougars harvested was positively associated with the number of verified complaints (rate ratio = 2.60413, *z* = 3.429, *P*<0.001). For each 100% increase in harvest of adults the odds of a verified complaint the following year increased by a factor of 2.6 or 160%. Similarly for each 10% increase in harvest, the odds of a verified complaint increased by 16%. The number of people residing in each GMU was also positively related to an increased number of verified complaints (rate ratio = 1.000001066, *z* = 2.285, *P* = 0.022). For each additional 10,000 people in an area the chance of a verified complaint being filed increased by a factor of 1.000001066 or less than 1%.

Two models (Table 6) were also selected for determining which factors may be related to the number of livestock depredations in each GMU. The first model was g(y) = −3.155876+0.428854 (number of cougars harvested) +0.038094 (total cougar population) –0.005630 (cougars harvested*total cougar population).

Both of the main effects were found to be significant in this model. Once again, the number of adult cougars harvested was positively related to the number of livestock depredations in each GMU (rate ratio = 1.5355, *z* = 5.097, *P*<0.001). For each adult harvested the odds of a depredation went up by 53%. The total expected cougar population was also found to be positively associated with the number of verified livestock depredations (rate ratio = 1.03883, *z* = 5.02, *P*<0.001), but for each additional cougar on the landscape the odds of subsequent depredation went up only 4%.

The second model was selected to determine which factors may influence livestock depredations was g(y) = −2.019+1.216(proportion of adult cougars harvested) +1.278×10^−6^ (human population) +2.248×10^−5^ (proportion of adult cougars harvested*human population).

Both main effects were statistically significant in this model. The proportion of adult cougars harvested was positively related to the number of livestock depredations (rate ratio = 3.37367, *z* = 3.186, *P* = 0.001). The human population in each GMU was also significantly positively related to increased livestock depredations (rate ratio = 1.000001278, *z* = 2.012, *P* = 0.044). For each 100% increase in harvest rate of cougars (removal of all adult animals) the odds increased by a factor of 3.4 or 240%. Similarly a 10% increase in proportion of adult cougars harvested increased the odds of a livestock depredation occurring the following year by 24%.

The final models were selected to determine which factors influenced the number of total depredations (large and small livestock) reported in each GMU (Table 6). The model was g(y) = −2.910767+0.386019 (number of cougars harvested) +0.038721 (total cougar population) –0.005189 (cougars harvested*total cougar population). The main effects in this model were significant and positively associated with the number of total depredations. The number of adult cougars harvested had a rate ratio of 1.47111 (*z* = 5.057, *P*<0.001) while the total cougar population had a rate ratio of 1.03948 (*z* = 5.716, *P*<0.001). Once again for each adult cougar harvested the odds of a depredation occurring the following year were 1.5 or increased by 50%.

The other model selected for total depredations was g(y) = −1.753+0.9633 (proportion of adult cougars harvested) +1.164×10^−6^ (human population) +2.206×10^−5^ (proportion of adult cougars harvested*human population).

All of the main effects were significant in this model. The proportion of adult cougars harvested was positively related to the number of total depredations (rate ratio = 2.62, *z* = 2.747, *P* = 0.006). For each 100% increase in adult cougar harvested the odds of a depredation occurring the following year increased by 162%. Similarly for each 10% increase in resident adult cougar harvest the odds of a depredation being filed the following year increase 16%. The human population in each GMU was also marginally associated with total depredations (rate ratio = 1.000001164, *z = *1.999, *P* = 0.045).

## Discussion

Bases on our results, we reject the “remedial hunting” hypothesis and support the “source-sink” hypothesis on effects of sport hunting on complaints and livestock depredations. There were several different factors that influence the number of cougar complaints and depredations across the state of Washington. In increasing order of importance these include: the human population, the number of livestock, number of cougars, the number of cougars killed, and proportion of cougars killed. Consistent with expectations, each additional cougar on the landscape increased the odds of a complaint or livestock depredation by about 5%. However, contrary to expectations, each additional cougar killed on the landscape increased the odds by about 50%, or an order of magnitude higher. By far, hunting of cougars had the greatest effects, but not as expected. Very heavy hunting (100% removal of resident adults in 1 year) increased the odds of complaints and depredations in year 2 by 150% to 340%. It appears that remedial sport hunting to reduce complaints and depredations is actually associated with increased, not decreased, complaints and depredations the following year.

Increased hunting fails to account for compensatory immigration and the shift in the sex-age structure towards younger cougars, which may be responsible for the increased reports and depredations [2], [15], [16].

Within Washington, Robinson et al. [15] found that heavy hunting (25% mortality) resulted in increased compensatory immigration with a resulting abundance of younger males. By contrast, Cooley et al. [16] found that light hunting (10% mortality) and no hunting resulted in compensatory emigration by young males and a stable older male structure in the population. In the same areas, Maletzke [30] found that heavy hunting resulted in a doubling of male cougar home range size and home range overlap. All else being equal, this doubling of home range size should double the number of human-occupied areas in each male cougar’s home range [30]. By the same token, each doubling of home range overlap should double the number of male cougars encountered by each human occupied area [30]. In addition, Kertson et al. [31], [32], [33] found that young cougars are more likely to be found in human-occupied areas then their older counterparts. Finally, Keehner [34] found that heavy hunting of cougars corresponded with females and kittens moving into sub-optimal habitats and killing sub-optimal prey species to avoid potentially infanticidal immigrant males. Elsewhere, Beier [35] found that juveniles and young adults may be responsible for the majority of the cougar-human conflicts in many areas and Torres et al. [36] found that male cougars are much more likely than females to engage in large livestock depredations. The above changes in sex/age structure and space-use by cougars following increased hunting could account for the observed increase in complaints and depredations in WA. We do not know which sex and age classes were responsible for the majority of complaints and depredations, but we do know that increased hunting was associated with increased, not decreased, complaints and depredations.

Our results are supported by a case study from two Washington cougar populations, where one was lightly hunted and one heavily hunted. The lightly hunted population (11±0.04 mortality rate) with a net male emigration rate of –12% [16], was located in Kittitas County (2478 mi^2^) with an average 38,842 people, 21,441 large livestock, and 138 cougars. Kittitas County had an average of 6.33 total complaints/year, 2.12 verified complaints/year, 0.66 livestock depredations/year and 0.83 total depredations/year (Table 7). The heavily hunted (0.24±0.07 mortality rate) population with a net male immigration rate of +11%, was located in Stevens County (2,297 mi^2^) and had 42,032 people, 22,293 large livestock and 207 cougars. Stevens County had an average number of 38.16 total complaints/year, 6.00 verified complaints/year, 2.66 livestock depredations/year, and 3.67 total depredations/year (Table 8). Stevens county had 1.5 times (50% more) as many cougars as Kittitas county, but had 3–6 times as many complaints and depredations. It appears the putative solution (heavy hunting) may have actually been exacerbating the problem in Stevens County.

**Table 7:** Reports filed in Kittitas County, Washington from January 2005–May 2010.

	Verified Reports	Total Reports	Livestock Depredations	Total Depredations
**2005**	5	11	1	1
**2006**	3	9	1	1
**2007**	0	1	0	0
**2008**	0	3	0	0
**2009**	4	10	2	2
**2010**	1	4	0	1

**Table 8:** Reports filed in Stevens County, Washington from January 2005–May 2010.

	Verified Reports	Total Reports	Livestock Depredations	Total Depredations
**2005**	5	50	2	3
**2006**	8	47	4	5
**2007**	8	21	2	3
**2008**	3	25	1	1
**2009**	3	41	2	2
**2010**	9	15	5	8

Remedial hunting of cougars, in Washington, was associated with increased, not decreased, complaints and depredations. We encourage other researchers to test for the efficacy of remedial hunting on other carnivore species such as black bears, brown bears, grizzly bears, jaguars, leopards, lions and tigers to see if the source-sink hypothesis generalizes to those species as well.

## Supporting Information

Figure S1 **Average number of reports filed by county from Jan. 2005–May 2010 in Washington.** Total reports, verified reports and verified livestock depredations averaged over the 5.5 year time frame (January 2005–May 2010) for each county in Washington.
(TIF)

Figure S2 **Average number of reports filed by GMU from Jan 2005–May 2010 in Washington.** Total reports, verified reports, and verified livestock depredations averaged over the 5.5 year time frame (January 2005–May 2010) for each GMU in Washington.
(TIF)

Results S1 **Statistical program R outputs.** Statistical program R outputs for all of the final models selected. Variables include: year (year2), cougar population (poptot), number of large livestock (livlarg), human population (humpop), the number of cougars harvested (hvst), and the proportion of adult cougars harvested (harvest_adlt).
(DOCX)
