# Supplementary figures and images for: Effects of Remedial Sport Hunting on Cougar Complaints and Livestock Depredations
Source: PLoS One. 2013 Nov 19;8(11):e79713. doi: 10.1371/journal.pone.0079713 (PMC3834330; doi:10.1371/journal.pone.0079713)

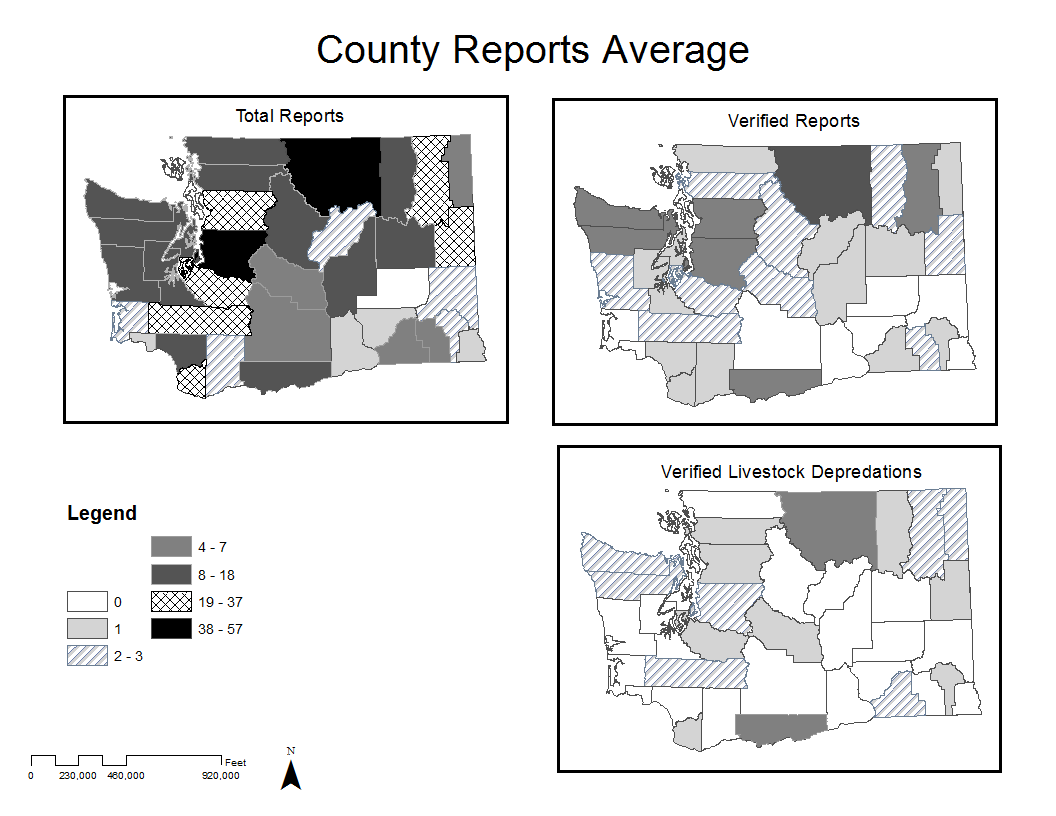

Supplement: Figure S1 — Average number of reports filed by county from Jan. 2005–May 2010 in Washington. Total reports, verified reports and verified livestock depredations averaged over the 5.5 year time frame (January 2005–May 2010) for each county in Washington. (TIF) [file pone.0079713.s001.tif]

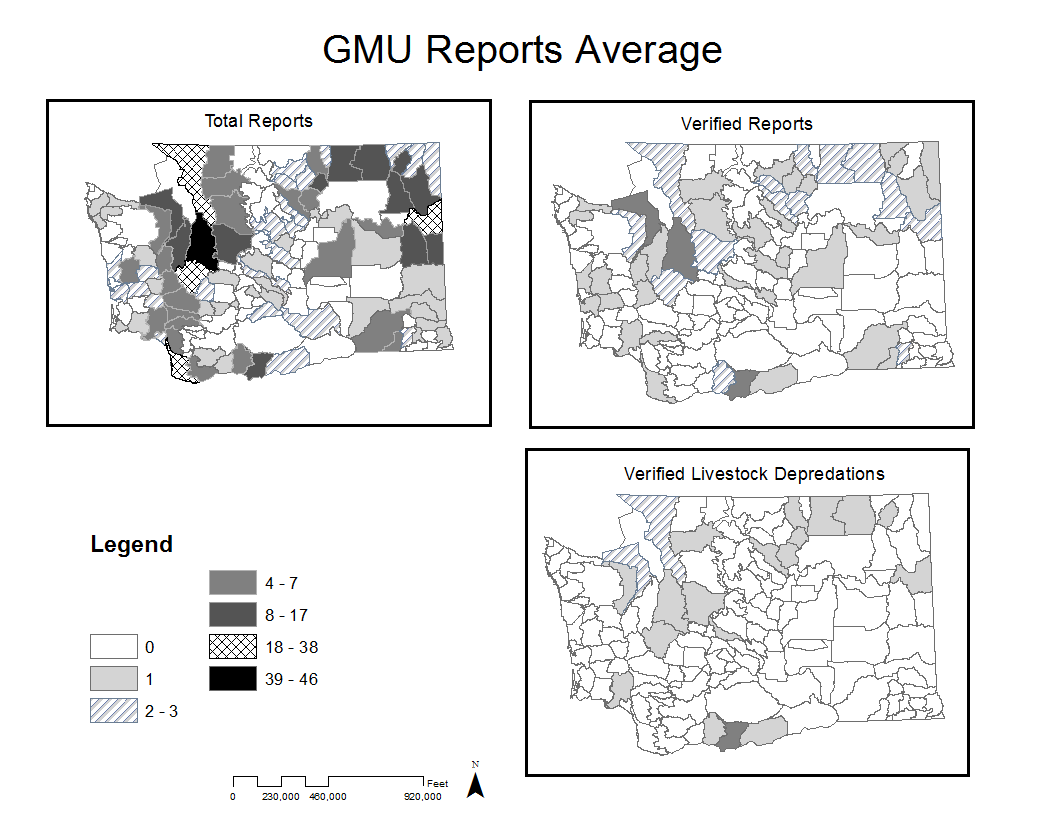

Supplement: Figure S2 — Average number of reports filed by GMU from Jan 2005–May 2010 in Washington. Total reports, verified reports, and verified livestock depredations averaged over the 5.5 year time frame (January 2005–May 2010) for each GMU in Washington. (TIF) [file pone.0079713.s002.tif]
