# Supplementary material for: Effects of Remedial Sport Hunting on Cougar Complaints and Livestock Depredations
Source: PLoS One. 2013 Nov 19;8(11):e79713. doi: 10.1371/journal.pone.0079713 (PMC3834330; doi:10.1371/journal.pone.0079713)
Supplement: Results S1 — Statistical program R outputs. Statistical program R outputs for all of the final models selected. Variables include: year (year2), cougar population (poptot), number of large livestock (livlarg), human population (humpop), the number of cougars harvested (hvst), and the proportion of adult cougars harvested (harvest_adlt). (DOCX) [file pone.0079713.s003.docx]

*Statistical Program R Outputs of Results*

*County Based Tests*

**Total Reports~ human population, total cougar population, large livestock**

Coefficients:

Estimate Std. Error z value Pr(>|z|)

(Intercept) -1.225e+00 4.605e-01 -2.660 0.00781 **

humpop 1.641e-02 3.125e-03 5.253 1.50e-07 ***

poptot 2.809e-02 3.965e-03 7.084 1.40e-12 ***

livelarg 1.458e-04 2.475e-05 5.892 3.81e-09 ***

humpop:poptot -1.334e-04 2.712e-05 -4.919 8.72e-07 ***

humpop:livelarg -7.117e-07 1.453e-07 -4.898 9.66e-07 ***

poptot:livelarg -1.010e-06 1.815e-07 -5.564 2.64e-08 ***

humpop:poptot:livelarg 5.973e-09 1.364e-09 4.379 1.19e-05 ***

---

(Dispersion parameter for Negative Binomial(0.8988) family taken to be 1)

Null deviance: 357.85 on 233 degrees of freedom

Residual deviance: 269.53 on 226 degrees of freedom

AIC: 1549.8

Number of Fisher Scoring iterations: 1

Theta: 0.8988

Std. Err.: 0.0946

2 x log-likelihood: -1531.7690

**Verified Reports~ year, total cougar population**

Coefficients:

Estimate Std. Error z value Pr(>|z|)

(Intercept) 0.2129444 0.3153187 0.675 0.49947

year2 -0.2480663 0.0874987 -2.835 0.00458 **

poptot 0.0084313 0.0022334 3.775 0.00016 ***

year2:poptot 0.0003281 0.0005979 0.549 0.58314

---

(Dispersion parameter for Negative Binomial(0.9624) family taken to be 1)

Null deviance: 337.30 on 233 degrees of freedom

Residual deviance: 228.09 on 230 degrees of freedom

AIC: 761.68

Number of Fisher Scoring iterations: 1

Theta: 0.962

Std. Err.: 0.178

2 x log-likelihood: -751.682

**Livestock Depredations~ human population, total cougar population, large livestock**

Coefficients:

Estimate Std. Error z value Pr(>|z|)

(Intercept) -5.050e+00 1.207e+00 -4.182 2.88e-05 ***

humpop 1.789e-02 7.895e-03 2.266 0.023473 *

poptot 4.363e-02 1.031e-02 4.230 2.33e-05 ***

livelarg 2.336e-04 6.203e-05 3.766 0.000166 ***

humpop:poptot -1.940e-04 6.396e-05 -3.032 0.002425 **

humpop:livelarg -1.317e-06 4.191e-07 -3.143 0.001673 **

poptot:livelarg -1.873e-06 4.991e-07 -3.753 0.000175 ***

humpop:poptot:livelarg 1.151e-08 3.399e-09 3.385 0.000712 ***

(Dispersion parameter for Negative Binomial(0.5881) family taken to be 1)

Null deviance: 226.31 on 233 degrees of freedom

Residual deviance: 162.28 on 226 degrees of freedom

AIC: 476.86

Number of Fisher Scoring iterations: 1

Theta: 0.588

Std. Err.: 0.139

2 x log-likelihood: -458.857

**Total Depredation~ human population, total cougar population, large livestock**

Coefficients:

Estimate Std. Error z value Pr(>|z|)

(Intercept) -4.629e+00 1.064e+00 -4.352 1.35e-05 ***

humpop 1.583e-02 7.016e-03 2.257 0.024038 *

poptot 4.137e-02 9.056e-03 4.568 4.92e-06 ***

livelarg 2.176e-04 5.407e-05 4.025 5.70e-05 ***

humpop:poptot -1.709e-04 5.644e-05 -3.029 0.002454 **

humpop:livelarg -1.195e-06 3.619e-07 -3.302 0.000961 ***

poptot:livelarg -1.707e-06 4.331e-07 -3.941 8.13e-05 ***

humpop:poptot:livelarg 1.013e-08 2.950e-09 3.433 0.000596 ***

(Dispersion parameter for Negative Binomial(0.7172) family taken to be 1)

Null deviance: 258.05 on 233 degrees of freedom

Residual deviance: 176.97 on 226 degrees of freedom

AIC: 533.53

Number of Fisher Scoring iterations: 1

Theta: 0.717

Std. Err.: 0.159

2 x log-likelihood: -515.527

*GMU Based Tests*

**Total Reports~ proportion of adult cougars harvested, human population**

Coefficients:

Estimate Std. Error z value Pr(>|z|)

(Intercept) 4.529e-01 7.115e-02 6.365 1.95e-10 ***

harvest_adlt 4.400e-01 2.259e-01 1.948 0.05141 .

Hum_Pop 3.788e-06 3.685e-07 10.280 < 2e-16 ***

harvest_adlt:Hum_Pop 1.925e-05 5.428e-06 3.547 0.00039 ***

---

Signif. codes: 0 ‘***’ 0.001 ‘**’ 0.01 ‘*’ 0.05 ‘.’ 0.1 ‘ ’ 1

(Dispersion parameter for Negative Binomial(0.4614) family taken to be 1)

Null deviance: 815.62 on 674 degrees of freedom

Residual deviance: 647.43 on 671 degrees of freedom

AIC: 2568.2

Number of Fisher Scoring iterations: 1

Theta: 0.4614

Std. Err.: 0.0378

2 x log-likelihood: -2558.2230

**Verified Reports~ number of cougars harvested, total cougar population**

Coefficients:

Estimate Std. Error z value Pr(>|z|)

(Intercept) -1.970170 0.172525 -11.420 < 2e-16 ***

hvst 0.308764 0.060773 5.081 3.76e-07 ***

poptot 0.031093 0.005343 5.819 5.92e-09 ***

hvst:poptot -0.003842 0.001173 -3.274 0.00106 **

(Dispersion parameter for Negative Binomial(0.4333) family taken to be 1)

Null deviance: 496.17 on 679 degrees of freedom

Residual deviance: 422.43 on 676 degrees of freedom

AIC: 1123.1

Number of Fisher Scoring iterations: 1

Theta: 0.4333

Std. Err.: 0.0697

2 x log-likelihood: -1113.0980

**Verified Reports~ proportion of adult cougars harvested, human population**

Coefficients:

Estimate Std. Error z value Pr(>|z|)

(Intercept) -1.081e+00 1.006e-01 -10.738 < 2e-16 ***

harvest_adlt 9.571e-01 2.791e-01 3.429 0.000606 ***

Hum_Pop 1.066e-06 4.668e-07 2.285 0.022340 *

harvest_adlt:Hum_Pop 1.453e-05 6.718e-06 2.163 0.030580 *

---

Signif. codes: 0 ‘***’ 0.001 ‘**’ 0.01 ‘*’ 0.05 ‘.’ 0.1 ‘ ’ 1

(Dispersion parameter for Negative Binomial(0.3399) family taken to be 1)

Null deviance: 444.32 on 674 degrees of freedom

Residual deviance: 416.63 on 671 degrees of freedom

AIC: 1157.1

Number of Fisher Scoring iterations: 1

Theta: 0.3399

Std. Err.: 0.0510

2 x log-likelihood: -1147.0810

**Livestock Depredations~ cougars harvested, total population of cougars**

Coefficients:

Estimate Std. Error z value Pr(>|z|)

(Intercept) -3.155876 0.264796 -11.918 < 2e-16 ***

hvst 0.428854 0.084144 5.097 3.46e-07 ***

poptot 0.038094 0.007586 5.02 2 5.12e-07 ***

hvst:poptot -0.005630 0.001659 -3.394 0.000689 ***

(Dispersion parameter for Negative Binomial(0.2555) family taken to be 1)

Null deviance: 310.00 on 679 degrees of freedom

Residual deviance: 253.63 on 676 degrees of freedom

AIC: 644.87

Number of Fisher Scoring iterations: 1

Theta: 0.2555

Std. Err.: 0.0561

2 x log-likelihood: -634.8710

**Livestock Depredations~ proportion of adult cougars harvested, human population**

Coefficients:

Estimate Std. Error z value Pr(>|z|)

(Intercept) -2.019e+00 1.466e-01 -13.772 < 2e-16 ***

harvest_adlt 1.216e+00 3.817e-01 3.186 0.00144 **

Hum_Pop 1.278e-06 6.348e-07 2.012 0.04417 *

harvest_adlt:Hum_Pop 2.248e-05 9.063e-06 2.480 0.01313 *

---

Signif. codes: 0 ‘***’ 0.001 ‘**’ 0.01 ‘*’ 0.05 ‘.’ 0.1 ‘ ’ 1

(Dispersion parameter for Negative Binomial(0.1848) family taken to be 1)

Null deviance: 268.75 on 674 degrees of freedom

Residual deviance: 247.24 on 671 degrees of freedom

AIC: 668.72

Number of Fisher Scoring iterations: 1

Theta: 0.1848

Std. Err.: 0.0377

Warning while fitting theta: alternation limit reached

2 x log-likelihood: -658.7200

**Total Depredations~ cougars harvested, total cougar population**

Coefficients:

Estimate Std. Error z value Pr(>|z|)

(Intercept) -2.910767 0.236055 -12.331 < 2e-16 ***

hvst 0.386019 0.076338 5.057 4.27e-07 ***

poptot 0.038721 0.006774 5.716 1.09e-08 ***

hvst:poptot -0.005189 0.001488 -3.488 0.000488 ***

(Dispersion parameter for Negative Binomial(0.3136) family taken to be 1)

Null deviance: 360.63 on 679 degrees of freedom

Residual deviance: 295.05 on 676 degrees of freedom

AIC: 743.66

Number of Fisher Scoring iterations: 1

Theta: 0.3136

Std. Err.: 0.0647

2 x log-likelihood: -733.6630

**Total Depredations~ proportion of adult cougars harvested, human population**

Coefficients:

Estimate Std. Error z value Pr(>|z|)

(Intercept) -1.753e+00 1.316e-01 -13.315 < 2e-16 ***

harvest_adlt 9.633e-01 3.506e-01 2.747 0.00601 **

Hum_Pop 1.164e-06 5.823e-07 1.999 0.04559 *

harvest_adlt:Hum_Pop 2.206e-05 8.306e-06 2.655 0.00792 **

---

Signif. codes: 0 ‘***’ 0.001 ‘**’ 0.01 ‘*’ 0.05 ‘.’ 0.1 ‘ ’ 1

(Dispersion parameter for Negative Binomial(0.2218) family taken to be 1)

Null deviance: 310.50 on 674 degrees of freedom

Residual deviance: 288.64 on 671 degrees of freedom

AIC: 775.32

Number of Fisher Scoring iterations: 1

Theta: 0.2218

Std. Err.: 0.0421

2 x log-likelihood: -765.3180
